# Alterations in the Ocular Surface Fungal Microbiome in Fungal Keratitis Patients

**DOI:** 10.3390/microorganisms7090309

**Published:** 2019-09-02

**Authors:** Gumpili Sai Prashanthi, Rajagopalaboopathi Jayasudha, Sama Kalyana Chakravarthy, Shalem Raj Padakandla, Chinthala Reddy SaiAbhilash, Savitri Sharma, Bhupesh Bagga, Somasheila I. Murthy, Prashant Garg, Sisinthy Shivaji

**Affiliations:** 1 Jhaveri Microbiology Centre, L.V. Prasad Eye Institute, Kallam Anji Reddy campus, Hyderabad, Telangana 500034, India; 2 Tej Kohli Cornea Institute, L.V. Prasad Eye Institute, Kallam Anji Reddy campus, Hyderabad, Telangana 500034, India

**Keywords:** fungal keratitis, ocular fungal microbiome, conjunctiva, cornea, NGS

## Abstract

Keratitis, an inflammatory disease of the eye, when neglected could lead to sight-threatening complications and ultimately blindness. Globally, over a million people are affected by keratitis annually. Keratitis has a microbial etiology and is caused by bacteria, fungi, viruses, etc. The present study compared the ocular surface fungal microbiome of healthy individuals and individuals with fungal keratitis. Fungal microbiomes from the conjunctival swabs of healthy individuals and from conjunctival swabs and corneal scrapings of individuals with fungal keratitis were generated using ITS2 region amplicons. Microbiomes were sequenced using Illumina MiSeq 2 × 250 base pair chemistry with a paired-end protocol. Based on Alpha diversity indices, phylum and genera level diversity, abundance differences, and heat map analysis, the fungal microbiomes of conjunctival swabs and corneal scrapings of individuals with fungal keratitis exhibited dysbiosis (alterations in the diversity and abundance) compared to the ocular surface microbiome of the healthy control individuals. This is the first report indicating dysbiosis in the fungal microbiome of conjunctival swabs and corneal scrapings in individuals with fungal keratitis. A total of 11 genera present in the majority of the eyes constituted the variable core ocular microbiome.

## 1. Introduction

Dysbiosis refers to the alteration, imbalance, or change in the composition of resident commensal microbial communities found in the gut of healthy individuals [1]. Over the years, dysbiosis in the human gut microbiome has been shown to be associated with auto-immune diseases (diabetes, rheumatoid arthritis, muscular dystrophy, multiple sclerosis and fibromyalgia), inflammatory diseases (obesity, enterocolitis, inflammatory bowel disease, and vaginosis) [2,3], cancers, and mental disorders including Alzheimer’s disease, anxiety, and autistic disorders [4]. In contrast, studies linking the gut microbiome with ocular diseases are relatively rare [5]. Horai et al. [6] and Huang et al. [7] were among the first to demonstrate that dysbiosis in the gut microbiome is associated with uveitis in mice and in uveitis patients respectively. This observation of gut microbiome dysbiosis in uveitis patients was further confirmed by several others [8,9] including in Behcet′s disease patients with uveitis [10] and in patients with ocular mucosal disease [11], as well as in age-related macular degeneration (AMD) in mice and in AMD patients [12,13]. In our recent studies, we demonstrated dysbiosis in gut bacterial and fungal microbiomes in patients with ocular inflammatory diseases like uveitis [8,9], bacterial keratitis [14], and fungal keratitis [15]. A question that has not been addressed is whether changes or dysbiosis in the ocular microbiome accompanies the diseased state of the eye. Conventional culture-based and PCR-based methods of conjunctival swabs and corneal scrapings did not yield fungi in > 80% of the samples analysed [16,17,18,19]. The common fungi in the culture positive ocular surface samples included *Alternaria* sp., *Fusarium* sp., *Aspergillus niger*, *Aspergillus flavus*, *Curvularia* sp., *Penicillium* sp., *Helminthosporium* sp., *Candida albicans*, *C. guilliermondii*, *C. parapsilosis*, *Saccharomyces cerevisiae*, *Hormodendrum* sp., and *Rhodotorula rubra*. In contrast, when the fungal microbiome of the ocular surface of healthy individuals was analysed by Next generation sequencing (NGS), using internal transcribed spacer 2 (ITS2) sequencing as a proxy for fungi, the method identified fungi in 73.5% swabs, detected 65 distinct genera with *Aspergillus*, *Setosphaeria*, *Malassezia*, and *Haematonectria* in all the studied eyes [19]. Further Alpha diversity in the two eyes was similar and sex had no effect, but Chao 1 and Simpson indices were altered by age [19]. Considering that fungi are the causative agents of several ocular diseases, such as keratitis, endophthalmitis, blepharitis [20], and conjunctivitis [21], it is important to establish the ocular surface fungal microbiome in the diseased state and compare it to that of the healthy control.

Keratitis is an inflammatory disease of the eye, which manifests as corneal opacity, redness, pain, and itchiness of the eye. Bacteria, fungi, viruses, or protozoa are known to cause keratitis. Keratitis, if neglected, could lead to sight-threatening complications like corneal scarring, perforation, endophthalmitis, and ultimately blindness [22,23]. Globally, over a million people are affected by keratitis annually. In India, nearly 2 million people are affected by keratitis annually [24,25]. The present study was undertaken to acquire data on the ocular surface fungal microbiome of healthy individuals and individuals with fungal keratitis by NGS to ascertain whether dysbiosis in the ocular microbiome of keratitis individuals compared to healthy controls is a feature of keratitis. The study also sheds light on whether the conjunctival swab microbiome is different from corneal microbiome in keratitis individuals.

## 2. Materials and Methods 

### 2.1. Recruitment of Subjects

Healthy controls (HC) (14 males and 11 females) in an age group that ranged from 23–77 years (mean 42.32 years) without any ocular pathology were recruited to analyse conjunctival surface microbiome (HC-SW). In addition 10 more individuals (6 males and 4 females) undergoing photorefractive keratectomy (PRK) and without any ocular disease in an age group of 21–28 years (mean 25 years) were also included as a separate group for analysing healthy corneal surface microbiome as the recovery of corneal epithelium from healthy individuals was not permitted by the institutional ethics committee. PRK is normally done to correct refractive error. From 35 individuals (20 males and 15 females) in an age group of 18–80 years (mean 48.3 years) with microbiologically-proven fungal keratitis, conjunctival swabs (FK-SW) were also included in the study. In addition, corneal scrapings (FK-CR) from these individuals were collected. Patients identified with bacterial, viral, or mixed infections were not included in the study. In the FK group, 21 out of 35 FK individuals at the time of presentation had received prior topical antifungals which they continued. All 21 patients had taken Natamycin. In addition to Natamycin, 4 patients had also taken Itraconazole (FK02), Voriconazole (FK26), and Fluconazole (FK22 and FK35). HC individuals had not received any antifungal medicine. The annual incidence of fungal keratitis in the Indian population is 3.4 per 10,000 [26]. Using the population proportion method with an 80% confidence interval and a 4% margin of error, the sample size derived was 35. Hence 35 patients with fungal keratitis were recruited in this study. Written informed consent was taken from all study participants prior to sample collection. The study was conducted in accordance with the Declaration of Helsinki and the study was approved by the institutional review board of L.V. Prasad Eye Institute, Hyderabad (Ethics Ref. No. LEC 06-14-060, 9 June 2014).

### 2.2. Sample Collection

A total of 50 conjunctival swabs from 25 healthy individuals (HC-SW) were obtained from both the right (RE) and left eye (LE). The conjunctival swabs were collected as described earlier (Shivaji et al. 2019). The same method was also used for the collection of conjunctival swab samples from FK patients (FK-SW) from only the infected eye. Corneal scrapings were also collected from FK patients (FK-CR) from the infected eye using sterile surgical blade No.15 fixed to a steel Bard Parker handle following the topical administration of proparacaine hydrochloride (0.5%) eye drops. Corneal epithelium from 10 patients undergoing PRK was also collected as a control for corneal scrapings from FK-CR. Corneal scraping and conjunctival swab samples collected for DNA extraction were stored at −80 °C until DNA extraction. 

### 2.3. Culturing of Fungi

Conjunctival swab and corneal scraping samples were smeared on a clean glass slide to detect fungi by the potassium hydroxide (10%) method and also cultured on several media such as 5% sheep blood agar, chocolate agar, Sabouraud dextrose agar, potato dextrose agar, brain heart infusion broth and thioglycollate broth without any inhibitory substances to identify the culturable fungi [27]. Fungal isolates were identified based on colony characteristics and microscopic features of the spores.

### 2.4. DNA Extraction

Conjunctival swabs and corneal scrapings were transferred to a sterile tube containing phosphate buffered saline (PBS, pH 7.4, Sigma-Aldrich, Bangalore, India) for DNA extraction. Genomic DNA was isolated using QIAamp DNA minikit (Qiagen, Hilden, North Rhine-Westphalia, Germany). Buccal swab and tissue spin protocols of Qiagen were followed for DNA isolation of conjunctival swab and corneal scraping samples respectively. Final DNA elution was performed with 30, 50, and 100 µL of AE buffer. It was observed that 30 µL was optimum for DNA extraction Unused sterile Isohelix swabs moistened with sterile PBS were processed as sample blanks for DNA extraction.

### 2.5. PCR Amplification, Illumina Library Preparation, and Amplicon Sequencing

ITS2, a region of the fungal ribosomal small subunit RNA was amplified with ITS3 (5′-GCATCGATGAAGAACGCAGC-3′) and ITS4 (5′-TCCTCCGCTTATTGATATGC-3′) primers [28] using the extracted DNA as described in Shivaji et al. [19]. All PCR reagents were prepared using sterile nuclease free water. Negative control (PCR reagents without template DNA) PCR amplification was carried out to exclude the possibility of false-positive PCR results due to contamination. PCR was consistently negative for DNA from Isohelix swabs, DNA extraction reagents, and PCR reagents. Sequencing also did not yield any fungal reads.

The fungal amplicon libraries were prepared according to standard Illumina protocol as discussed by Dehingia et al. [29]. Sequencing of libraries was done using Illumina MiSeq 2 × 250 base pair chemistry with a paired-end protocol at Xcelris Genomics Pvt. Ltd., Ahmedabad, India.

### 2.6. Taxonomy Assignment of Sequenced Reads

Paired-end reads of each sample were assembled using FLASH software [30]. Low quality sequences (average Phred score < 25) and chimeric sequences were removed with Prinseq-lite [31] and Usearch61 respectively [32]. With high quality reads, operational taxonomic unit (OTU) picking was performed using an `open reference operational taxonomic unit (OTU) picking′ approach in the Quantitative Insights into Microbial Ecology (QIIME) pipeline [33] using UNITE OTUs (ITS) 12.11 (alpha release) clustered at a 97% sequence similarity. Taxonomic assignments for denovo-OTUs were obtained using a Wang Classifier [34] with a bootstrap of 80%. OTUs containing < 0.001% of the total number of high quality reads (sparse OTUS) were removed for further analysis.

### 2.7. Diversity Analyses of the Microbiomes

Rarefaction curves and Alpha diversity indices (Shannon diversity, Simpson index, number of observed OTUs, and Chao1 index) of the microbiomes were plotted using R-Vegan 2.4-2 package (http://vegan.r-forge.r-project.org/). Consequently, a t-test was performed to analyse whether alpha diversity was significantly different between the 3 groups.

### 2.8. Identification of Differentially Abundant Genera

Differentially abundant fungal genera that were associated between HC-SW, FK-SW, and FK-CR were determined by a Wilcoxon signed rank test, BH (Benjamini Hochberg) corrected *p* < 0.05. A two-dimensional heat map, with the rank-normalised abundances (scaled between 0 and 1) of the differentially abundant fungal genera, was plotted in R. 

### 2.9. Principal Coordinate Analysis of the Microbiomes of Conjunctival Swabs and Corneal Scrapings of Fungal Keratitis Patients Who Have Taken Antifungal Medication

Wilcoxon test was performed between fungal keratitis patients who had taken antifungal medications (FK-SW-T and FK-CR-T) and compared with those who did not take antifungal medications (FK-SW-UT and FK-CR-UT). Subsequently, principal coordinate analysis (PCoA) graphs based on JSD (Jensen–Shannon divergence) distances with OTU abundances were generated between FK-T vs. FK-UT. 

### 2.10. Interaction Networks between Fungal Genera in the Microbiomes 

Separate interaction networks were generated based on pair-wise correlations between abundances of different fungal genera in the 3 cohorts of microbiomes using CoNet [35] in Cytoscape [36]. Spearman correlation coefficient (r) was used to obtain the pair-wise correlations between abundances of the fungal genera.

### 2.11. Correlation of Fungal Genera in HC-SW, FK-SW, and FK-CR

Correlation analysis of fungal microbiomes was performed with genera having a median abundance of > 0.0001 by Spearman’s rank correlation. Plots were derived by Corrplot package in R. 

## 3. Results

### 3.1. Sample Details 

The data reported in the present study includes analyses of ocular fungal microbiomes from conjunctival swabs of healthy controls (HC, *n* = 25), corneal epithelium from patients undergoing PRK (*n* = 10), conjunctival swabs from fungal keratitis patients (FK-SW, *n* = 35), and corneal scrapings from fungal keratitis patients (FK-CR, *n* = 35) (Table S1). Demographic and the fungal microbiome data of a subset of HC (*n* = 17 out of 25) was published by us earlier [19]. Please also note that corneal epithelium from patients undergoing PRK (*n* = 10) served as a control for scrapings from FK-CR who exhibited a growth on the cornea.

### 3.2. Detection of Fungi by Culturable Approach

The conjunctival swabs of healthy controls and corneal scrapings of FK individuals were analysed for the presence of fungi by the culturable approach. The results indicated fungi in 3 out of 50 conjunctival swabs of HC (6%) and in 35 out of 35 corneal scrapings (FK-CR, 100%). The fungi in HC predominantly belonged to the genus *Aspergillus* and were identified as *Aspergillus flavus* (RE001 and RE028) and *Aspergillus niger* (LE002), whereas in the corneal scrapings (FK-CR) they were identified as *Aspergillus flavus*, *Aspergillus fumigatus*, *Fusarium* sp., *Fusarium solani*, *Colletotrichum coccodes*, *Exserohilum rostratum*, *Cladosporium* sp., *Curvularia lunata*, as well as several unidentified septate fungal filaments, aseptate fungal filaments, and Dematiaceous fungus (Table S1) based on microscopy. The conjunctival swab of FK patients were not analysed for culturable fungi because we were permitted by the Institutional Ethics Committee to take only one conjunctival swab as required for microbiome preparation. 

### 3.3. NGS Analysis of the Fungal Ocular Microbiomes 

In the 25 healthy controls, a total of 50 conjunctival swab samples from both the eyes were processed for microbiome library preparation out of which only 37 samples (17 RE and 20 LE) yielded ITS2 amplicons and were used for Illumina sequencing. In addition, conjunctival swabs and corneal scrapings from 35 FK patients were also processed for the generation of ITS2 microbiomes out of which microbiomes were generated only from 23 FK-SW and 29 FK-CR samples. The remaining samples in HC-SW, FK-SW, FK-CR, and the corneal epithelium from patients undergoing PRK (*n* = 10) were negative for the amplification of ITS2 following PCR and were not used for library preparation. This may be attributed to the low fungal load in the sample or the presence of PCR inhibitors. It is also possible that the absence of fungi in PRK patients may also be attributed to the fact that these patients were treated with povidone-iodine 5 minutes prior to the surgery. Following Illumina sequencing, the 37 HC-SW samples together yielded 15.46 million high-quality reads (after the removal of chimeric sequences and reads with a mean Phred score less than 25) and the high quality reads per microbiome ranged from 88897 to 1.371 million reads with an average of 0.41 million reads per microbiome (Table 1 and Table S2). Compared to HC-SW, the 23 FK-SW reads ranged from 61695 to 0.88 million reads with an average of 0.46 million reads per microbiome and in the 29 FK-CR microbiomes, the reads ranged from 153623 to 0.98 million reads with an average of 0.37 million reads per microbiome (Table 1). All 89 microbiomes after QC were used for the analyses. The reads of the three cohorts did not exhibit any significant difference (*p* = 0.436). All microbiomes from HC-SW, FK-SW, and FK-CR were matched for age (*p* = 0.158) and gender (*p* = 1.0) and were from Telangana and Andhra Pradesh, two states in the southern part of India. 

### 3.4. OTU Analysis 

In the above microbiomes, the percentage of high quality reads in a microbiome that could be assigned to an OTU ranged from a minimum of 76.64% to a maximum of 99.93% reads (at 97% sequence identity) (Table S2). Sparse OTUs (representing 0.001% of the total HQ reads) were not considered for further analysis. In the three cohorts, a total of 1084 OTUs were identified out of which 340 were reference OTUs and 744 were denovo-OTUs (Table S3). The observed increase in the number of denovo-OTUs compared to the reference OTUs would imply a limited number of available sequenced fungal organisms in the database. Rarefaction curves of HC-SW, FK-SW, and FK-CR showed a tendency to plateau, indicating a reasonable sequencing depth and coverage for the sequenced samples (Figure S1).

### 3.5. Alpha Diversity Indices of the Ocular Fungal Microbiomes 

**Figure 1:**
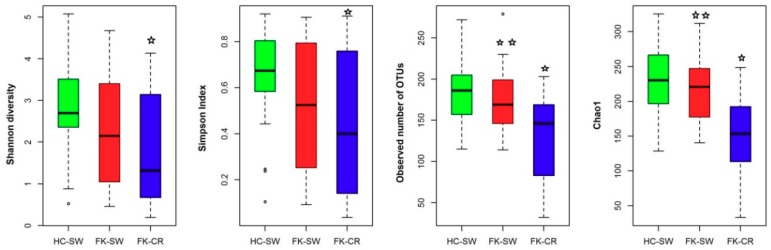
Alpha diversity indices of the ocular fungal microbiomes from conjunctival swabs of healthy controls (HC-SW, *n* = 37), conjunctival swabs of fungal keratitis patients (FK-SW, *n* = 23), and corneal scraping of fungal keratitis patients (FK-CR, *n* = 29). ✩ indicates significant difference between HC-SW and FK-CR by Student’s t-test. ✩✩ indicates significant difference between FK-SW and FK-CR by Student′s t-test.

Analysis of the Alpha diversity indices indicated that the Shannon diversity index (*p* = 0.011), Simpson index (Evenness) (*p* = 0.013), Observed number of OTUs (*p* = 0.001), and Chao1 index (Richness) (*p* = 0.001) were statistically significant when compared across all the three cohorts (HC-SW, FK-SW, and FK-CR) using the Kruskal Wallis test. However when we compared the indices between two groups, it was observed that HC-SW and FK-SW were not significantly different (Student′s t-test) whereas when HC-SW was compared with FK-CR, all indices were significantly different. Furthermore, when FK-SW and FK-CR were compared there were significant difference observed in two indices, namely the observed number of OTUs and Chao1 (Figure 1). Thus the results indicated that the fungal community varied significantly in HC-SW compared to FK–CR respectively (Figure 1).

### 3.6. Fungal Community Composition of the Ocular Microbiomes

**Figure 2:**
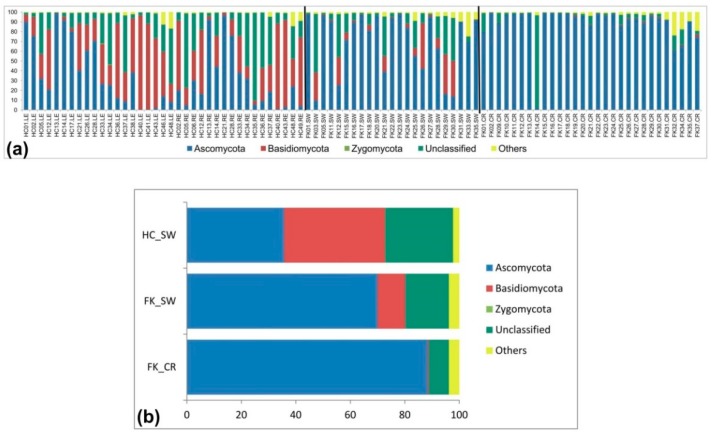
Abundance (%) (**a**) and average abundance (%) of fungal phyla (**b**) from microbiomes of conjunctival swabs of healthy controls (HC-SW, *n* = 37), conjunctival swabs of fungal keratitis patients (FK-SW, *n* = 23), and corneal scrapings of fungal keratitis patients (FK-CR, *n* = 29).

Assignment of the OTUs was attempted both at the phylum and genus level. In HC-SW, Ascomycota (mean abundance, 35.66%) and Basidiomycota (mean abundance, 37.05%) were the two dominant phyla and together accounted for approximately 80% of the total abundance. In contrast, Zygomycota was a minor phylum (mean abundance, 0.05%). The remaining reads could not be assigned to a known phylum and were included as unclassified (24.96%) and others (2.28%) which included singletons and sparse OTUs with < 0.001% of total high-quality reads (Figure 2a,b, Table 2 and Table S3). Ascomycota was the most dominant phylum in FK-SW (mean abundance, 69.99%) and in FK-CR (mean abundance, 88.36%) and when compared to HC-SW, the abundance was significantly increased (*p* = 0.001) in both whereas the abundance of Basidiomycota was significantly decreased in FK-SW (mean abundance, 10.12%) and FK-CR (mean abundance, 0.35%) compared to HC-SW (*p* = 0.001). In contrast, Zygomycota was detected in very few eyes and abundance was low (Figure 2a,b and Table 2). Unclassified and others were also detected in FK-SW and FK-CR.

**Figure 3:**
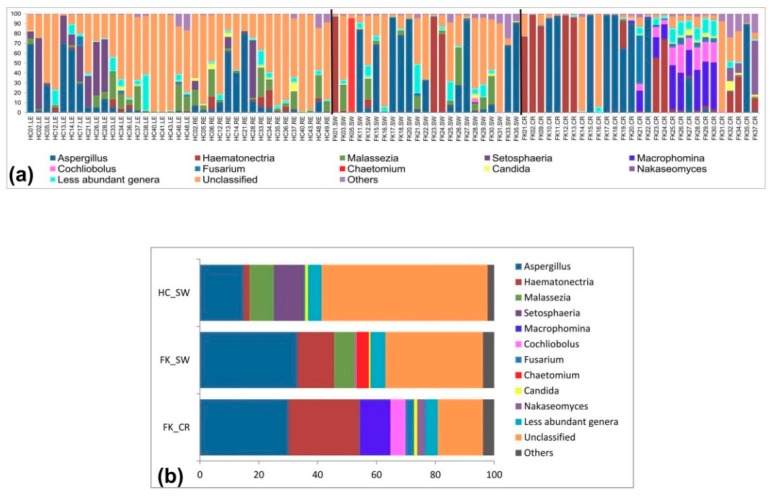
Abundance (%) (**a**) and average abundance (%) of fungal genera (**b**) from microbiomes of conjunctival swabs of healthy controls (HC-SW, *n* = 37), conjunctival swabs of fungal keratitis patients (FK-SW, *n* = 23), and corneal scrapings of fungal keratitis patients (FK-CR, *n* = 29).

At the genus level, only 62.31% of the reads (23138204/37131168) across all three cohorts (HC-SW, FK-SW, and FK-CR) could be assigned to 111 different genera and the remaining reads were assigned as unclassified (33.99%) genera and others (3.7%) (Figure 3a,b). *Aspergillus* was the only genus present in all 89 sampled eyes and its abundance ranged from 0.002% to a maximum of 98.86% (Table S4). The other genera present in more than 70% of the eyes (26 out of 37 sampled eyes) in HC-SW included *Malassezia*, *Emericella*, *Haematonectria*, *Candida*, *Cladosporium*, *Fusarium*, *Choiromyces*, and *Cochliobolus*. Seven of these genera, namely *Malassezia*, *Haematonectria*, *Candida*, *Cladosporium*, *Fusarium*, *Choiromyces*, and *Cochliobolus* were equally abundant in FK-SW. In contrast, in FK-CR *Haematonectria*, *Fusarium*, and *Cochliobolus* were the only three genera present in > 70% of the eyes that were sampled (Table S4). The remaining 102 genera were present in 0 to 25 of the eyes in the three cohorts and their abundance varied (Table S4). 

**Figure 4:**
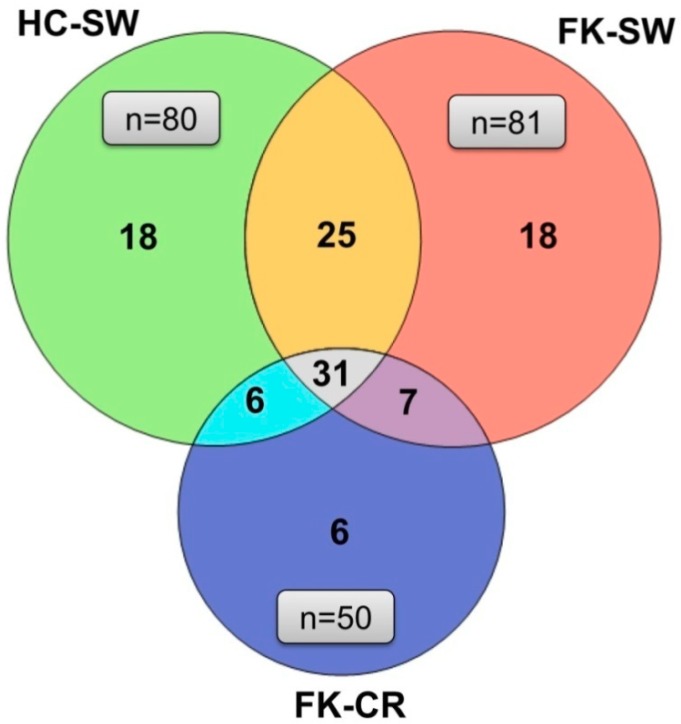
Venn diagram representing fungal genera present exclusively in each group (HC-SW, FK-SW, and FK-CR) and common between all three groups and two groups.

**Figure 5:**
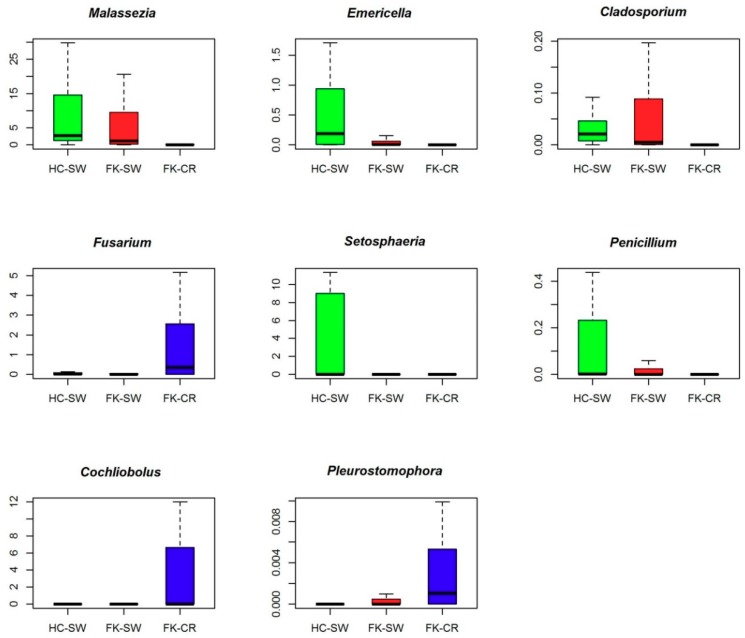
Fungal genera exhibiting significant (BH corrected *p* < 0.05) differential abundance in ocular fungal microbiomes of healthy controls (HC-SW, *n* = 37) compared to microbiomes of fungal keratitis swabs and corneal scrapings (FK-SW, *n* = 23 and FK-CR, *n* = 29). Differentially abundant genera having a median abundance of > 0.001% in at least one group of samples have been depicted. Median abundances (horizontal line) and interquartile ranges have been indicated in the plots.

The Venn diagram (Figure 4) shows the distribution of the 111 different genera between HC-SW, FK-SW, and FK-CR. It was observed that 56 and 37 genera were shared between HC-SW and FK-SW and HC-SW and FK-CR respectively and 38 genera between FK-CR and FK-SW. Table S5a shows the genera that are commonly shared between all three groups (HC-SW, FK-SW, and FK-CR) and those that are shared between two groups (HC-SW and FK-SW, HC-SW and FK-CR, and FK-SW and FK-CR). In addition, the genera which are unique to a particular group are shown in Table S5b. We also observed that three genera could discriminate between HC-SW and FK-SW, 33 genera between HC-SW and FK-CR, and 27 genera between FK-SW and FK-CR (Table S6a–c). Figure 5 shows boxplots of eight different genera discriminating between the three cohorts. It was observed that the median abundance of the genera *Malassezia*, *Emericella*, *Cladosporium*, *Setosphaeria*, and *Penicillium* decreased in FK-SW and FK-CR compared to HC-SW whereas *Fusarium*, *Cochliobolus*, and *Pleurostomophora* increased in FK-CR compared to HC-SW and FK-SW.

**Figure 6:**
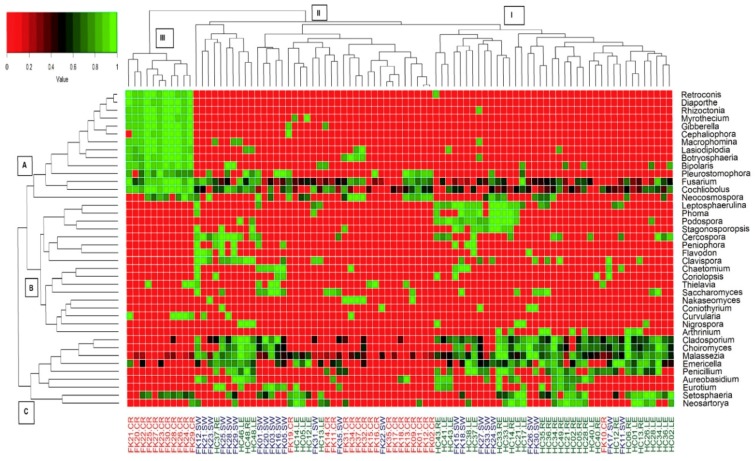
Two dimensional heat map showing rank normalised abundances (scaled between 0 and 1) of 40 differentially abundant fungal genera in the ocular microbiomes of healthy controls conjunctival swabs (HC-SW, *n* = 37), conjunctival swabs of fungal keratitis patients (FK-SW, *n* = 23), and corneal scraping of fungal keratitis patients (FK-CR, *n* = 29). Differentially abundant genera having a median abundance of > 0.0001% in at least one group of samples have been depicted. The discriminating genera have been arranged along the two dimensions (axes) based on hierarchical clustering.

A heat map is a graphical representation of data to indicate the level of abundance using colours. In our study, it was used to represent the abundance of 40 discriminating genera based on rank normalised abundances across the samples. Consequently, hierarchical clustering was also performed to show clustering of the samples. Heat map analysis (Figure 6) indicated that HC-SW, FK-SW, and FK-CR could be separated in three clusters I, II, and III (Figure 6). Majority of HC-SW microbiomes were in cluster I, whereas FK-CR was distributed into cluster II and III, and only 11 FK-CR were present in cluster III. FK–SW did not form a separate cluster and nine and 14 microbiomes were distributed in clusters I and II respectively. The differential clustering could be attributed to the genera as indicated in the heat map (Figure 6). For instance, cluster III of FK-CR was due to a greater abundance of 14 genera (group A) consisting of *Retroconis*, *Diaporthe*, *Rhizoctonia*, *Myrothecium*, *Gibberella*, *Cephaliophora*, *Macrophomina*, *Lasiodiplodia*, *Botryosphaeria*, *Bipolaris*, *Pleurostomophora*, *Fusarium*, *Cochliobolus*, and *Neocosmospora* which were present in FK-CR and absent in the other two cohorts. FK-CR also differed from HC-SW and FK-SW due to a lower abundance or absence of nine other genera namely *Cladosporium*, *Choiromyces*, *Malassezia*, *Emericella*, *Penicillium*, *Aureobasidium*, *Eurotium*, *Setosphaeria*, and *Neosartorya* (group C). Seventeen other genera (*Leptosphaerulina*, *Phoma*, *Podospora*, *Stagonosporopsis*, *Cercospora*, *Peniophora*, *Flavodon*, *Clavispora*, *Chaetomium*, *Coriolopsis*, *Thielavia*, *Saccharomyces*, *Nakaseomyces*, *Coniothyrium*, *Curvularia*, *Nigrospora*, and *Arthrinium)* were distributed more in HC-SW and FK-SW compared to FK-CR (Figure 6).

### 3.7. Principal Coordinate Analysis of the Microbiomes of Conjunctival Swabs and Corneal Scrapings of Fungal Keratitis Patients Who Have Taken Antifungal Medication 

A Wilcoxon test between FK-SW-T vs. FK-SW-UT and FK-CR-T vs. FK-CR-UT did not show any significant difference (*p* < 0.05). PCoA (Figure S2) based on JSD distances between OTU abundances in the fungal microbiomes of conjunctival swabs (FK-SW-T vs. FK-SW-UT) and corneal scrapings (FK-CR-T vs. FK-CR-UT) of fungal keratitis patients indicated that a majority of the microbiomes in both the groups (87% i.e., 20 out of 23 in FK-SW and 76% i.e., 22 out of 29 in FK-CR) formed a single cluster.

### 3.8. Interactions between Fungal Groups Inhabiting the Ocular Surface of Healthy Subjects and FK Patients 

**Figure 7:**
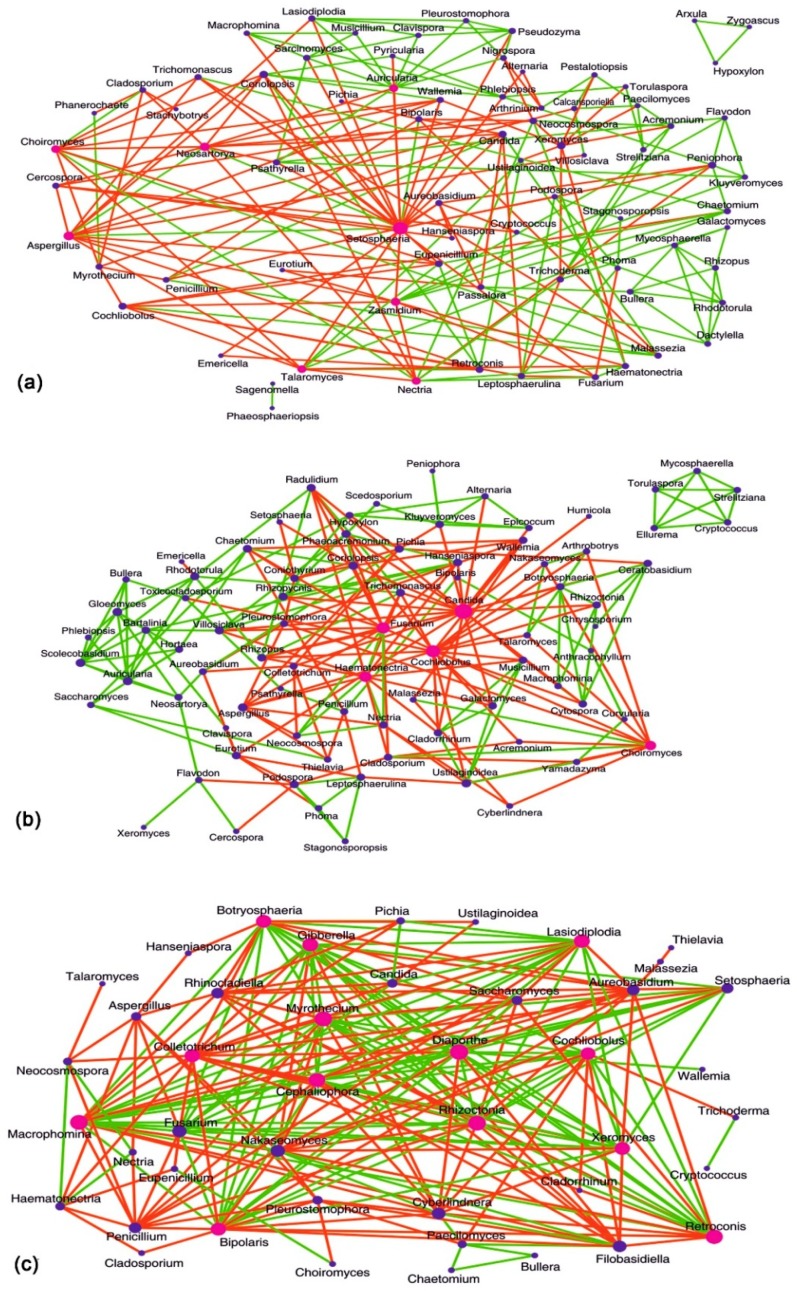
Fungal interaction network in the conjunctival microbiome of healthy (HC-SW) controls (**a**), conjunctival microbiome of fungal keratitis (FK-SW) patients (**b**) and corneal microbiome of fungal keratitis (FK-CR) patients (**c**) showing significant co-occurrence and co-exclusion relationships at the genera level. Each node represents a genus and the sizes of the nodes in the network correspond to their degree of interaction. The hub genera (with > 10 interactions) have been highlighted with pink nodes. The positive and negative correlations/associations have been indicated with green and red edges respectively.

Three interaction networks (HC-SW, FK-SW, and FK-CR) were generated based on pair-wise correlations between abundances of different fungal genera. In all three interaction networks (HC-SW, FK-SW, and FK-CR), a single, large, well-connected network of fungal genera was observed (Figure 7). In addition in HC-SW and FK-SW, minor networks were also observed (Figure 7a,b). In all networks, certain ′hub′ genera (with > 10 interactions) could be identified. In HC-SW, eight hub genera namely *Setosphaeria*, *Aspergillus*, *Neosartorya*, *Zasmidium*, *Auricularia*, *Talaromyces*, *Nectria*, and *Choiromyces* were detected and all of them exhibited both positive and negative interactions. *Setosphaeria* exhibited 13% positive interaction whereas *Auricularia* exhibited 80% positive interaction (8 of 10 genera). *Setosphaeria* was the largest hub genera in HC-SW and interacted negatively with pathogenic genera like *Candida*, *Lasiodiplodia*, *Alternaria*, *Aureobasidium*, etc. In FK-SW, five hub genera were detected and included pathogens like *Candida*, *Cochliobolus*, *Fusarium*, *Haematonectria*, and *Choiromyces*. Two of these hub genera *Candida* and *Cochliobolus* exhibited only negative interactions with 23 and 17 genera respectively (Figure 7b) and the genera they interacted with were plant pathogens such as *Botryosphaeria*, *Colletotrichum*, *Rhizoctonia*, *Ceratobasidium*, *Cytospora*, *Epicoccum*, and *Galactomyces*, or human pathogen like *Acremonium*, *Eurotium*, *Talaromyces*, and *Trichomonascus*. As compared to HC-SW and FK-SW, a maximum number of 13 hub genera were detected in FK-CR. These hub genera included plant pathogens like *Diaporthe*, *Gibberella*, *Macrophomina*, *Myrothecium*, *Lasiodiplodia*, *Bipolaris*, *Cochliobolus*, *Rhizoctonia*, *Colletotrichum*, *Botryosphaeria*, *Retroconis*, *Cephaliophora*, and *Xeromyces*. The hub genera *Colletotrichum* was the only genus that exhibited overall negative interactions with 17 other genera whereas others exhibited both positive and negative interactions (Figure 7c).

### 3.9. Correlation of Fungal Genera in HC-SW, FK-SW, and FK-CR

**Figure 8:**
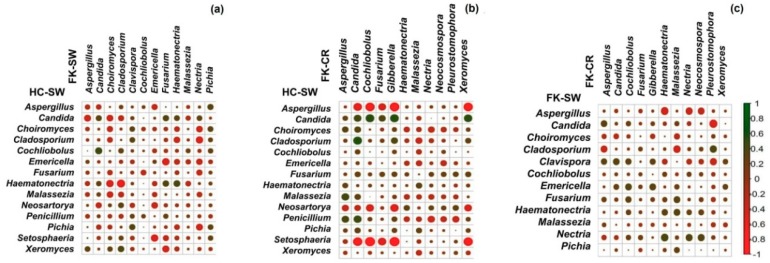
Correlation analysis (Spearman correlation) of fungal genera having a median abundance of >0.0001% in the microbiome of conjunctiva of healthy individuals (HC-SW) and fungal keratitis patients (FK-SW) (**a**), microbiome of conjunctiva of healthy individuals (HC-SW) and cornea of fungal keratitis patients (FK-CR) (**b**), and microbiome of conjunctiva (FK-SW) and cornea (FK-CR) of fungal keratitis patients (**c**).

Correlation of the fungal genera between HC-SW and FK-SW based on abundance and Spearman′s rank correlation coefficients (Figure 8a) indicated that *Emericella*, *Fusarium*, and *Malassezia* disclosed a negative correlation with the remaining 11 genera in FK-SW. In contrast, when correlation was monitored between HC-SW and FK-CR, the genera *Emericella* exhibited an overall negative correlation, *Fusarium* exhibited an overall positive correlation, whereas *Malassezia* showed both positive and negative correlations (Figure 8b). When analysis was done between FK-SW and FK-CR, the correlation was very distinct and none of the genera showed an overall positive or negative correlation (Figure 8c). A negative correlation of genera between HC-SW and FK-SW (Figure 8a) and HC-SW and FK-CR (Figure 8b) were higher than FK-SW and FK-CR (Figure 8c).

## 4. Discussion

Abelson et al. [37] in a brief review discussed the possibility of the eye having its own microbiome and concluded, ′The placid ocular surface is actually teeming with life that might hold the secret to treating ophthalmic disease′. Rightly so, St Leger et al. [38] established a resident commensal bacterial microbiome on the ocular surface of mice and identified *Corynebacterium mastitidis*, as a commensal, that protected the eye from *Candida albicans* and *Pseudomonas aeruginosa* infection. In a very recent study Ge et al. [39] investigated the ocular surface bacterial microbiome profile of healthy individuals and individuals with keratitis and reported an altered conjunctival microbiome in keratitis individuals. In an earlier study, they had demonstrated that in healthy individuals *Corynebacterium*, *Pseudomonas*, *Staphylococcus*, *Acinetobacter*, *Streptococcus*, *Millisia*, *Anaerococcus*, *Finegoldia*, *Simonsiella*, and *Veillonella* accounted for over 76% of the microbial community, possibly representing the “core genera” in normal conjunctival microbiota [40]. Similar studies on the ocular surface fungal microbiome and its relevance to ocular diseases like keratitis, uveitis, and blepharitis were lacking. Furthermore, microbiome changes that occur at the site of infection may also be associated with the disease phenotype. With this in mind, this study was undertaken to ascertain whether ocular fungal microbiome dysbiosis is associated with fungal keratitis.

The standard of eye care for infections like keratitis has always been to detect bacteria or fungi by culturing the microbe from the swab or corneal scraping. Repeatedly, the culture-based and the PCR-based methods have not yielded any fungi [16,17,18,41,42] and at best fungi were detected in about 12.5% of ocular surface swabs, whereas NGS identified fungi in 73.5% of the conjunctival swabs [19]. In the present study, we confirmed that fungi could be cultured from a small fraction of the healthy control swabs (only 6% from HC-SW). In contrast, all the corneal scrapings from FK-CR were positive for fungi. In HC-SW, *Aspergillus flavus* and *Aspergillus niger* were the only fungi identified whereas in the corneal scrapings (FK-CR), in addition to *Aspergillus flavus*, we identified *Aspergillus fumigatus*, *Fusarium* sp., *Fusarium solani*, *Colletotrichum coccodes*, *Exserohilum rostratum*, *Cladosporium* sp. *Curvularia lunata*, as well as several unidentified septate and aseptate fungal filaments and Dematiaceous fungi (Table S1). These observations are in agreement with a review which had compiled all articles published between 1950–2012 on the identity of microorganisms implicated in keratitis and listed 144 species of fungi from 92 genera [43]. Our results are in agreement with the above studies and have identified a total of 111 genera in this single study with many of them also present in the conjunctival swabs and corneal scrapings of FK patients.

Implicating a microbiome in the pathogenesis of a disease has been best studied in immune-mediated diseases [44], inflammatory diseases [2,3], cancers, and mental disorders [4], ocular diseases like uveitis [6,7,8,9,10,45], ocular mucosal disease [11], age-related macular degeneration [13], as well as bacterial and fungal keratitis [14,15]. These findings suggest a prime role for the gut bacterial microbiome in human health and disease irrespective of the disease location in the human body [5]. In the present study, a comparison of the fungal microbiomes in HC-SW, FK-SW, and FK-CR indicated that fungal phyla exhibited a difference in abundance with the increase in Ascomycota and decrease in Basidiomycota in FK-SW and FK-CR compared to HC-SW (Table 2). Furthermore, the four Alpha diversity indices indicated that diversity, evenness, number of observed OTUs, and richness significantly decreased in FK-CR, compared to HC-SW and two indices (number of observed OTUs and Chao1 index for richness) were significantly decreased in FK-CR compared to FK-SW. Thus, based on the differences in the abundance at the phyla level and differences in alpha diversity indices it appears that FK-SW and FK-CR were different compared to HC-SW (Figure 1), implying dysbiosis in the ocular fungal microbiome of keratitis patients. 

HC-SW microbiomes were associated with 80 distinct genera with 11 genera namely *Aspergillus*, *Setosphaeria*, *Malassezia*, *Haematonectria*, *Candida*, *Emericella*, *Penicillium*, *Fusarium*, *Cladosporium*, *Choiromyces*, and *Cochliobolus* present in a majority of the eyes (25 to 37 out of 37 eyes). This confirms our earlier study which had also indicated that with the exception of *Cochliobolus*, the other ten genera were present on the ocular surface of a majority of the eyes [19]. Based on the criteria of Turnbaugh et al. [46] it would appear that these 11 genera constitute the variable microbiome. Defining a core ocular fungal microbiome is also complicated by the fact that a significant number of OTUs both at the phylum and genera level were unidentified, implying that the available ITS2 fungal database needs to be strengthened. 

A comparison of the fungal microbiomes, at the genera level, further established the difference in the ocular surface microbiomes of the healthy controls (HC-SW) versus the eyes diseased with keratitis (FK-SW and FK-CR) (Figure 4). Both HC-SW and FK-SW with 80 genera each shared 56 genera implying that FK-SW microbiome is related to HC-SW. In fact, this is further strengthened by the observation that only three genera exhibited a significant differential abundance between HC-SW and FK-SW microbiomes (Table S6a). In contrast, FK-CR shared only 37 genera with HC-SW (Figure 4) and 33 genera differed in differential abundance (Table S6b). Heat map analysis of the 40 differential abundant genera also clearly separated HC-SW and FK-CR into different clusters. However, FK-SW microbiomes were affiliated to both, with 14 of the 23 FK-SW microbiomes forming a cluster with FK-CR and nine microbiomes with HC-SW microbiomes (Figure 6). We had earlier shown that fungal microbiomes were dominated either by Ascomycota or Basidiomycota [19] and we did not understand why one phyla existed at the exclusion of the other. In this study we observed that 11 microbiomes of FK-CR formed cluster III due to the presence of 14 genera, namely *Retroconis*, *Diaporthe*, *Rhizoctonia*, *Myrothecium*, *Gibberella*, *Cephaliophora*, *Macrophomina*, *Lasiodiplodia*, *Botryosphaeria*, *Bipolaris*, *Pleurostomophora*, *Fusarium*, *Cochliobolus*, and *Neocosmospora*. An interesting feature is that 13 out of the 14 genera were affiliated to Ascomycota except genus *Rhizoctonia* which is affiliated to Basidiomycota. It is of interest that in 23 microbiomes of FK-SW and the remaining 18 microbiomes of FK-CR the genera affiliated to Ascomycota were either under represented or absent. 

Based on the similarity and the unique genera associated with HC-SW, FK-SW, and FK-CR, it could be predicted that those genera that are shared between HC-SW and FK-SW and HC-SW and FK-CR are opportunistic pathogens, whereas the seven genera shared between FK-SW and FK-CR are pathogenic (Table S5a). In fact, 14 out of the 18 unique genera in HC-SW are either plant or animal pathogens. In Table S5b, it is clear that the unique genera in FK-SW and FK-CR are, as anticipated, opportunistic pathogens or pathogens (Table S5b).

Over the years, dysbiosis (alterations in the diversity and abundance) in the human gut microbiome has been shown to be associated with several diseases. Compared to the gut microbiome studies, ocular microbiome studies in normal and individuals with ocular disease using NGS have been neglected. Changes in diversity and abundance in the conjunctival bacterial microbiomes have been observed in patients with trachomatous disease caused by the bacterium *Chlamydia trachomatis* which causes conjunctival scarring [47], in the eyelash and tear samples from blepharitis patients [48], in the conjunctiva of contact lens wearers prone to inflammation [49] and individuals with dry eyes [42]. All these studies monitored the bacterial microbiomes of the eye. This is the first report on the observed dysbiosis in the fungal microbiome of conjunctival swabs and corneal scrapings in individuals with fungal keratitis compared to HC-SW. 

Interaction networks were generated to assess the interactions between the fungal genera in the community. A common feature shared by all the networks was that genera exhibited positive, negative, or both positive and negative interactions respectively. *Setosphaeria* was the largest hub genera in HC-SW interacting negatively with 20 other nodes and positively only with a few genera like *Neosartorya*, *Bipolaris*, and *Aspergillus*. *Setosphaeria* is a common plant pathogen and an opportunistic human pathogen causing sinusitis, keratitis, CNS vasculitis, and mycoses [50]. It probably plays an important role in inhibiting the growth of other pathogens like *Pseudozyma*, *Peniophora*, *Trichomonascus*, *Talaromyces*, etc. In FK-SW, five hub genera were detected and included pathogens like *Candida*, *Cochliobolus*, *Fusarium*, *Haematonectria*, and *Choiromyces*. Two of these genera *Candida* and *Cochliobolus* exhibited only negative interactions (Figure 7b) with plant pathogens like *Botryosphaeria*, *Colletotrichum*, *Rhizoctonia*, *Ceratobasidium*, *Cytospora*, *Epicoccum*, and *Galactomyces*, or human pathogen like *Acremonium*. The interaction was also observed with several yeasts which include *Cyberlindnera*, *Nakaseomyces*, and *Hanseniaspora* which are used as flavouring agents for fermentation or involved in food spoilage. A total of 12 unique hub genera were detected in FK-CR. These included plant pathogens like *Diaporthe*, *Macrophomina*, *Rhizoctonia*, *Myrothecium*, *Retroconis*, *Cephaliophora*, *Lasiodiplodia*, *Gibberella*, *Colletotrichum*, *Bipolaris*, *Botryosphaeria*, and *Xeromyces*. Only the hub genus *Colletotrichum* exhibited overall negative interactions with 17 other genera, whereas the others exhibited both positive and negative interactions. Thus it would appear that the hub genera in HC-SW would inhibit the other pathogens and at the same time positively regulate beneficial fungi. If this reasoning is correct, it is difficult to understand why *Setosphaeria* would positively regulate *Neosartorya*, *Bipolaris*, and *Aspergillus* which are pathogens. It is also difficult to comprehend as to why the hub genera in FK-SW and FK-CR exhibit both positive and negative interactions with other genera.

Correlation of the fungal genera between HC-SW and FK-SW based on abundance and Spearman’s correlation coefficients (Figure 8a) indicated that the genera *Emericella*, *Fusarium*, and *Malassezia* in HC-SW exhibited negative correlation with the remaining 11 genera in FK-SW. When the correlation was studied between HC-SW and FK-CR, one of the above genera namely *Emericella* and two other genera *Aspergillus* and *Setosphaeria* in HC-SW exhibited overall negative correlation with all the genera of FK-CR (Figure 8b). These results imply that these five genera in HC-SW are likely to be opportunistic pathogens/pathogens (*Emericella*, *Fusarium*, *Malassezia*, *Aspergillus*, and *Setosphaeria*) and play an important role in regulating the genera in FK-SW and FK-CR. It is surprising that we did not find any genera in FK-SW and FK-CR having overall either positive or negative interactions with the genera in HC-SW. Srinivasan et al. [51] suggested that strong positive correlations are predictive of metabolic or other dependencies whereas those that are negatively correlated may compete for nutrients or change in the environment in ways that inhibit the growth of each other. It is intriguing that *Fusarium* in HC-SW, which exhibited an overall negative correlation with FK-SW genera, exhibited an overall positive interaction with the same genera in FK-CR. To the best of our knowledge, this is the first study attempting to understand the interaction between the fungi associated with microbiomes from the conjunctiva of healthy individuals and the conjunctiva and corneal scraping of fungal keratitis patients. Correlation of the fungal genera in the above microbiomes was also inferred. Though the data was inferred, it does provide an insight into the positive and negative interactions operating simultaneously on the surface of the healthy and diseased eye.

## 5. Conclusions

This is the first report on the observed dysbiosis (alterations in the diversity and abundance) in the fungal microbiome of conjunctival swabs and corneal scrapings in individuals with fungal keratitis compared to HC-SW. Alteration in the fungal microbiota was observed both at the phylum and genera level. The ocular microbiome analysis indicated that 11 genera namely *Aspergillus*, *Setosphaeria*, *Malassezia*, *Haematonectria*, *Candida*, *Emericella*, *Penicillium*, *Fusarium*, *Cladosporium*, *Choiromyces*, and *Cochliobolus* present in majority of the eyes constituted the variable microbiome.

## Figures and Tables

**Table 1:** High quality reads from 89 ocular fungal microbiomes from conjunctival swabs of healthy controls (HC-SW, *n* = 37), conjunctival swabs of fungal keratitis patients (FK-SW, *n* = 23), and corneal scrapings of fungal keratitis patients (FK-CR, *n* = 29).

HQ Reads	HC-SW	FK-SW	FK-CR
	(*n* = 37)	(*n* = 23)	(*n* = 29)
Total	15,465,381	10,756,288	10,909,499
Maximum	1,371,325	883,303	980,760
Minimum	88,897	61,695	153,623
Average	417,983.3	467,664.7	376,189.6

**Table 2:** Mean abundance (%) of fungal phyla in the 89 ocular fungal microbiomes from conjunctival swabs of healthy controls (HC-SW, *n* = 37), conjunctival swabs of fungal keratitis patients (FK-SW, *n* = 23), and corneal scrapings of fungal keratitis patients (FK-CR, *n* = 29).

		HC-SW			FK-SW			FK-CR			*p*-Value			
Sl. No.	Phylum	Mean	Range	Present out of 37 Samples	Mean	Range	Present out of 23 Samples	Mean	Range	Present out of 29 Samples	HC-SW vs. FK-SW vs. FK-CR	HC-SW vs. FK-SW	HC–SW vs. FK-CR	FK-SW vs. FK-CR
1	Ascomycota	35.66	0.5–98.78	37	69.99	9.86–98.23	23	88.36	2.22–99.24	29	0.001	0.001	0.001	0.006
2	Basidiomycota	37.05	0.34–95.75	37	10.12	0.01–46.11	23	0.35	0–2.15	23	0.001	0.001	0.001	0.001
3	Zygomycota	0.05	0–0.98	2	0	0–0.07	1	0	0–0	0	0.461	0.845	0.215	0.278
4	Unclassified	24.96	0.54–90.22	37	16.07	0.09–60.17	23	7.47	0–94.56	29	0.001	0.012	0.001	0.006
5	Others *	2.28	0.29–16.88	37	3.82	0.71–24.93	23	3.82	0.38–23.84	29	NA	NA	NA	NA

* Includes singletons and sparse operational taxonomic units (OTUs) (with < 0.001% of total high quality reads).

## Supplementary Materials

The following are available online at https://www.mdpi.com/2076-2607/7/9/309/s1, **Figure S1:** Rarefaction curves of the ocular surface fungal microbiomes of (a) corneal scrapings of fungal keratitis patients (FK-CR, *n* = 29), (b) conjunctival swabs of fungal keratitis (FK-SW, *n* = 23), and (c) conjunctival swabs of healthy controls (HC-SW, *n* = 37). **Figure S2:** Principal coordinate analysis (PCoA) based on JSD (Jensen–Shannon divergence) distances between OTU abundances in the fungal microbiomes of conjunctiva (**a**) and corneal scrapings (**b**) of fungal keratitis patients who have taken antifungal medications (FK-SW-T and FK-CR-T, black), and who did not take antifungal medications (FK-SW-UT and FK-CR-UT, red).
