# Supplementary figures and images for: Alterations in the Ocular Surface Fungal Microbiome in Fungal Keratitis Patients
Source: Microorganisms. 2019 Sep 2;7(9):309. doi: 10.3390/microorganisms7090309 (PMC6780152; doi:10.3390/microorganisms7090309)

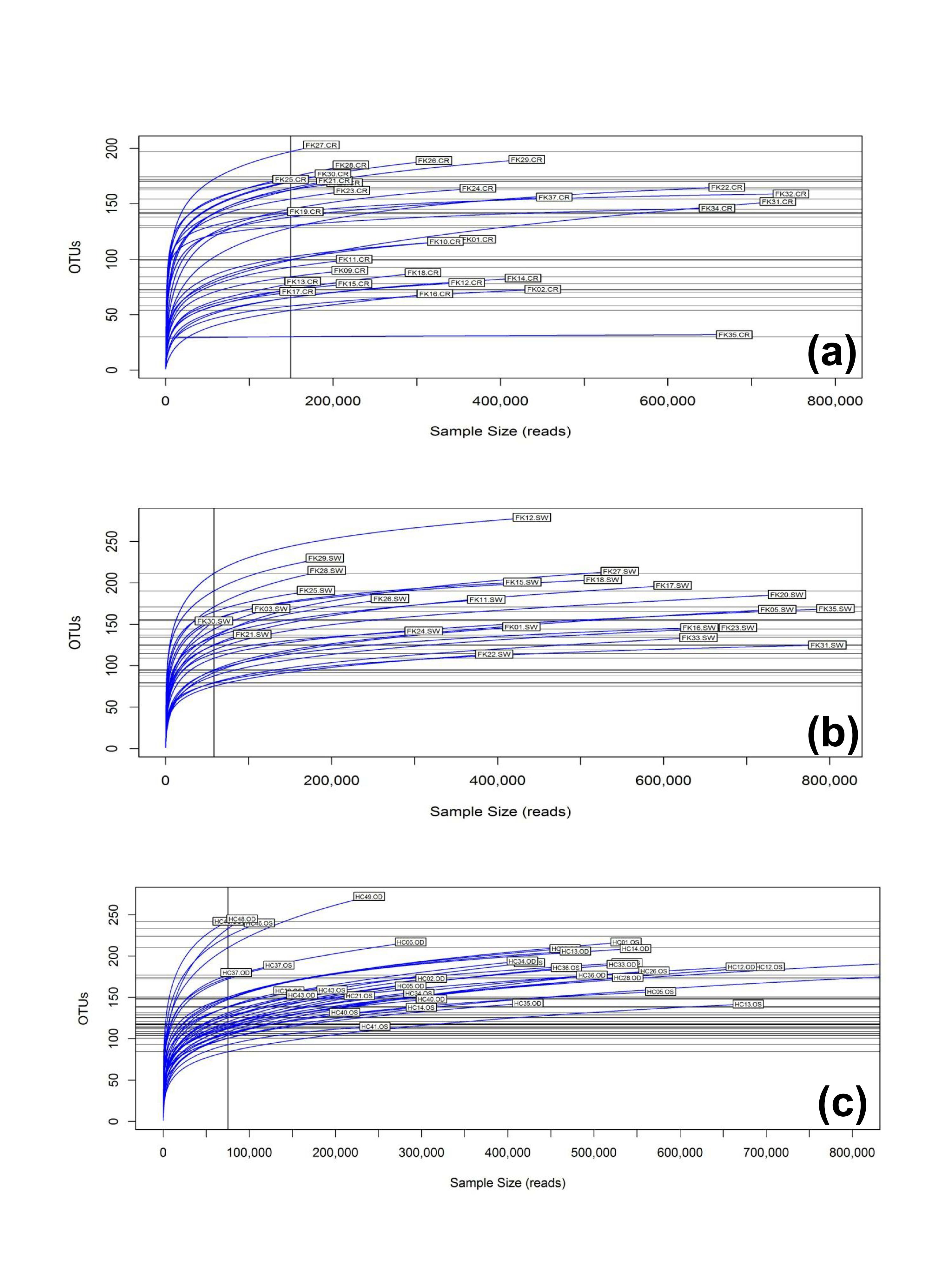

Supplement: Supplementary file 1 [file microorganisms-07-00309-s001.zip › Supplementary materials/Figure S1.jpg]

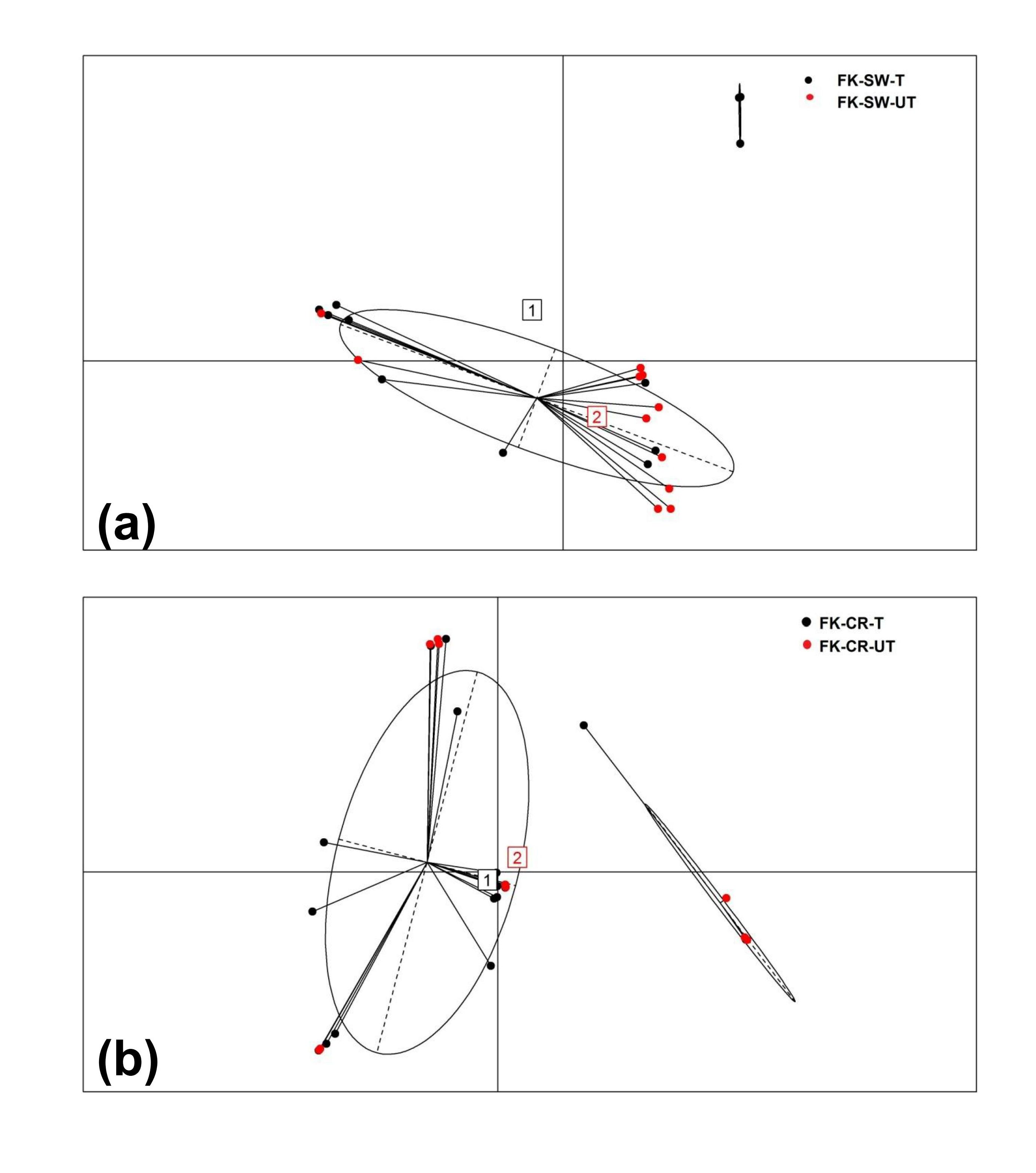

Supplement: Supplementary file 1 [file microorganisms-07-00309-s001.zip › Supplementary materials/Figure S2.jpg]
